# Immune Regulation of TNFAIP3 in Psoriasis through Its Association with Th1 and Th17 Cell Differentiation and p38 Activation

**DOI:** 10.1155/2020/5980190

**Published:** 2020-03-21

**Authors:** Yanyun Jiang, Wenming Wang, Xiaofeng Zheng, Hongzhong Jin

**Affiliations:** ^1^Department of Dermatology, Peking Union Medical College Hospital, Chinese Academy of Medical Sciences and Peking Union Medical College, Beijing, China; ^2^National Clinical Research Center for Dermatologic and Immunologic Diseases, Beijing, China

## Abstract

**Background:**

Psoriasis is an immune-mediated chronic inflammatory skin disorder in which the dysregulation of immune cells plays an important role in its development. Tumor necrosis factor- (TNF-) *α* antagonists affect the immune repertoire, while TNF-*α*-induced protein 3 (TNFAIP3) has a protective role against the deleterious effects of inflammation and participates in immune regulation.

**Objective:**

We investigated the immune regulation of *TNFAIP3* in the pathogenesis of psoriasis and determined whether it is involved in the antipsoriatic effect of TNF-*α* antagonists.

**Methods:**

mRNA levels were evaluated in blood from patients with moderate-to-severe psoriasis. The effects of TNF-*α* antagonists were examined in a mouse imiquimod- (IMQ-) induced psoriasis-like dermatitis model. In the mouse model, *TNFAIP3* mRNA expression was determined using RT-PCR. Serum levels of IL-17A, IL-23, IFN-*γ*, TNF-*α*, phosphorylated ERK1/2, p38, and JNK were measured using ELISA. The proportion of Th1 and Th17 cells in mouse spleens was analyzed using flow cytometry.

**Results:**

mRNA expression levels of *TNFAIP3* in the blood were significantly lower in patients with moderate and severe psoriasis (mean ± SD = 0.44 ± 0.25) compared with normal subjects (mean ± SD = 1.00 ± 0.82) (*P* < 0.01). In the mouse model, IMQ downregulated *TNFAIP3* expression levels, which were increased after TNF-*α* antagonist treatment (*P* < 0.05). Serum levels of Th17 cytokines (IL-17A and IL-23) and Th1 cytokines (IFN-*γ* and TNF-*α*) were significantly higher in the IMQ and IMQ/rat IgG1 groups compared with the control group, and the application of TNF-*α* antagonists significantly decreased the levels of inflammatory cytokines (*P* < 0.01). Notably, phosphorylated p38 levels were increased in the IMQ and IMQ/rat IgG1 groups compared with the control group but were downregulated by treatment with TNF-*α* antagonists (*P* < 0.05). Th1 and Th17 cells were significantly increased in the IMQ group compared with the control group (*P* < 0.01).

**Conclusion:**

*TNFAIP3* downregulation associated with Th1 and Th17 cell differentiation and p38 activation might contribute in part to the mechanism of immune dysfunction in psoriasis. TNF-*α* antagonists might partly exert their effects on psoriasis via this pathway.

## 1. Introduction

Psoriasis is a chronic inflammatory skin disease characterized by the hyperproliferation of keratinocytes and infiltration of inflammatory cells. It affects about 2% of the population worldwide, but the rate varies according to region [1]. The molecular mechanisms of psoriasis are complicated and unclear, but many studies have indicated that psoriasis is an immune-mediated disease. Th1 cells, Th17 cells, and their cytokines contribute to the pathogenesis of psoriasis [2, 3]. The number of Th17 cells and their associated secreted cytokines including IL-17A, IL-17F, and IL-22 was significantly elevated in the skin lesions and peripheral blood of patients with psoriasis [3]. Excessive activation of the MAPK (p38, JNK, and ERK1/2) signaling pathway has a key role in regulating the production of inflammatory mediators in psoriasis [4], and recently, p38 was shown to control IL-17 expression in mouse CD4^+^ T cells [5]. Therefore, the p38 signaling pathway might be related to T helper cell differentiation in psoriasis.

The tumor necrosis factor alpha-induced protein 3 (*TNFAIP3*) gene encodes the TNFAIP3 protein, also known as TNF-*α*-inducible zinc finger protein A20, a cytoplasmic zinc-finger protein that acts as a negative immunoregulatory protein under inflammatory states [6]. A previous study showed that the selective deletion of *TNFAIP3* in mice led to systemic inflammation under homeostatic conditions and the exacerbation of inflammatory skin disorders [7]. Of note, the *TNFAIP3* mRNA expression levels in peripheral blood mononuclear cells (PBMCs) from patients with psoriasis were negatively correlated with disease severity in psoriasis vulgaris [8]. The TNFAIP3 protein was initially identified as a negative feedback factor in the NF-*κ*B signaling pathway [6]. Moreover, it inhibited the expression of many proinflammatory cytokines including IL-17 in response to inflammatory stimuli [9]. *TNFAIP3* overexpression in keratinocytes significantly repressed inflammatory cytokines and chemokines [10]. These results indicated that TNFAIP3 is involved in the pathogenesis of psoriasis and might regulate inflammation through immune pathways associated with T helper cell differentiation and their related cytokines.

For the treatment of psoriasis, TNF-*α* antagonists block the effects of TNF-*α*, a pathogenic cytokine. However, the mechanism involved is complex and diverse, although previous studies suggested that TNF-*α* antagonists might affect intracellular signaling pathways resulting in a rapid reduction in the number of cells at inflammatory sites [11]. Furthermore, TNF-*α* antagonists were reported to disrupt Th1, Th17, and Th22 pathways, resulting in the clinical improvement of psoriasis [12]. In addition, *TNFAIP3* gene polymorphisms were associated with responses to TNF antagonists in psoriasis [13]. However, the relationship between the expression level of *TNFAIP3* and the biological function of TNF-*α* antagonists remains unclear.

Therefore, this study investigated the immune regulatory effects of *TNFAIP3* in psoriasis. We detected the relative mRNA expression levels of *TNFAIP3* in psoriatic patients. We also used an IMQ-induced psoriasis-like dermatitis model, a widely used animal model of psoriasis that closely resembles human psoriatic lesions, which is critically dependent on Th1- and Th17-associated proinflammatory cytokines [14]. We investigated the relative expressions of *TNFAIP3*, T helper cell differentiation, inflammatory cytokine secretions, and immune pathway activation in the IMQ-induced psoriasis-like dermatitis model. The immunomodulatory effects of TNF-*α* antagonists were also examined.

## 2. Materials and Methods

### 2.1. Ethics Statement

Human psoriasis studies were approved by the Ethical Committee of Peking Union Medical College Hospital. All animal experiments were approved by the Animal Care and Research Advisory Committee in the Institute of Laboratory Animal Sciences, Chinese Academy of Medical Sciences.

### 2.2. Patients and Controls

This study was conducted on 23 moderate-to-severe psoriatic vulgaris (PV) patients with psoriasis area and severity index (PASI) ≥ 10, who attended the outpatient clinic at the Dermatology Department of Peking Union Medical College Hospital from April 2019 to July 2019. All patients had received no antipsoriatic treatment in the past 4 weeks before the study started. Patient exclusion criteria included pregnancy or the presence of autoimmune diseases or major systemic diseases. Patients diagnosed with erythrodermic psoriasis, pustular psoriasis, or psoriatic arthritis were also excluded from the study. Twenty-five healthy controls (HCs) matched for age and gender were enrolled as a control group. Written consent, clinical information, and blood samples were obtained from all subjects.

### 2.3. Experimental Animals

BALB/c mice at 7–8 weeks of age (male, 20 ± 2 g) were purchased from Huafukang Company (Beijing, China). All mice were kept under specific pathogen-free conditions with a 12 h light-dark cycle. They were provided with a standard laboratory diet and water.

### 2.4. Animal Model of Psoriasis

Mouse anti-mouse TNF*α* Ab (clone #XT3.11) and control rat IgG1 were purchased from Bioxcell (West Lebanon, NH), and 5% imiquimod cream (Aldara) was purchased from 3M Pharmaceuticals (United Kingdom). IMQ-induced psoriasiform skin inflammation was induced in mice as described previously [15]. Briefly, hair on the backs of BALB/c mice was treated with a skin hair remover (Veet, China). The mice were randomly divided into four groups (*n* = 6–8 per group): control group, IMQ group, IMQ/rat IgG1 group, and IMQ/anti-TNF-*α* group. A daily topical dose of 62.5 mg of 5% imiquimod cream or control Vaseline was applied to the shaved dorsal skin of the mice for six consecutive days. Mice in the IMQ/rat IgG1 group and IMQ/anti-TNF-*α* group were intraperitoneally injected with 200 *μ*g anti-TNF-*α* antibody or control rat IgG1 1 day before (day −1) and 3 days after the imiquimod treatment (day 3). Mice were humanely euthanized on day 7 followed by sample collection. The severity of mouse psoriasis-like skin lesions was evaluated using a modified human scoring system PASI. The modified PASI system includes measurements for erythema, scaling, and thickening. Three parameters were scored independently on a scale from 0 to 4 (0: none; 1: slight; 2: moderate; 3: marked; and 4: highly marked). The cumulative dermatitis score was used to determine the severity of inflammation. Skin samples from different groups were collected and fixed in 4% formalin for 48 h. Tissue samples were dehydrated and then embedded in paraffin. Paraffin sections (4 *μ*m thickness) were stained with hematoxylin and eosin (H&E) for histopathology.

### 2.5. RNA and cDNA Preparations from PBMCs of Humans and Mice

Blood samples of humans and mice were diluted 1 : 1 with phosphate-buffered saline (PBS), and PBMCs were separated by density gradient centrifugation (Solarbio, Beijing, China). Total RNA was extracted from PBMCs using TRIzol reagent (Life Technologies, Grand Island, NY) according to the manufacturer's instructions. RNA concentration and quality were measured by a NanoDrop spectrophotometer. Samples with an A260/A280 ratio between 1.8 and 2.0 were used for further study. Then, reverse transcription for cDNA was performed using the PrimeScript RT Master Mix (Takara Bio Inc., Otsu, Japan).

### 2.6. Real-Time Quantitative PCR (RT-PCR) in Humans and Mice

RT-PCR was performed using the StepOnePlus™ Real-Time PCR System (Applied Biosystems, Foster City, CA) with SYBR Premix Ex Taq II (Takara Bio Inc.) in accordance with the instructions of the manufacturer. Primer sequences were as follows: human TNFAIP3 (forward: 5′-TGCTGCCCTAGAAGTACAATAGGAA-3′, reverse: 5′-GCAGCTGGTTGAGTTTATGCAAG-3′); human GAPDH (forward: 5′-TCGGAGTCAACGGATTTGGT-3′, reverse: 5′-TTCCCGTTCTCAGCCTTGAC-3′); mouse TNFAIP3 (forward: 5′-AGAACAGCTGAGATCAAGCCA-3′, reverse: 5′-GTGCTGGCACTCCATACAGA-3′); and mouse GAPDH (forward: 5′-AGGTCGGTGTGAACGGATTTG-3′, reverse: 5′-TGTAGACCATGTAGTTGAGGTCA-3′). Amplification reactions were performed using the following program: 95°C for 60 s followed by 40 cycles of 95°C for 30 s, 60°C for 30 s, and 72°C for 30 s, then a melting curve analysis from 60°C to 95°C. The relative TNFAIP3 expression level was normalized to GAPDH housekeeping mRNA transcripts using the 2^−*ΔΔ*Ct^ method. Each reaction was set up in at least duplicate wells.

### 2.7. Measurement of Cytokines and Phosphorylation Levels of ERK1/2, p38, and JNK

Mouse serum was obtained by centrifugation at 1000 × g for 20 min at 4°C and was collected and stored at −80°C until analysis. The concentrations of IL-17A, IL-23, IFN-*γ*, and TNF-*α* were measured by ELISA in accordance with the manufacturer's instructions (Mianmei, Jiangsu, China). Phosphorylation levels of ERK1/2, p38 and JNK were measured by ELISA (Mianmei, Jiangsu, China).

### 2.8. Flow Cytometry

Single-cell suspensions prepared from mouse spleens were incubated with a cell activation cocktail (BioLegend, San Diego, CA, USA) at 37°C and 5% CO_2_ for 6 h. Then, cells were stained for cell surface and intracellular markers with the following conjugated monoclonal antibodies: FITC anti-mouse CD3 (cat# 100203), PerCP/Cyanine5.5 anti-mouse CD8a (cat# 100734), APC anti-mouse IFN-*γ* (cat# 505810), and PE anti-mouse IL-17A (cat# 506904). All antibodies were purchased from BioLegend. CD3^+^CD8a^−^IL-17A^+^ cells were identified as Th17 cells, and CD3^+^CD8a^−^IFN-*γ*^+^ cells were identified as Th1 cells. Cells were analyzed with a BD Accuri C6 instrument (BD Biosciences) and FlowJo software v10.

### 2.9. Statistical Analysis

The results are presented as the mean ± SD. The two-tailed Student *t*-test was used for differences between the two groups, and one-way analysis of variance (ANOVA) was used for the four groups. All statistical analyses were performed with SPSS 23.0 software, and a level of *P* < 0.05 was considered statistically significant.

## 3. Results

### 3.1. *TNFAIP3* mRNA Expression in Patients with Psoriasis

**Figure 1:**
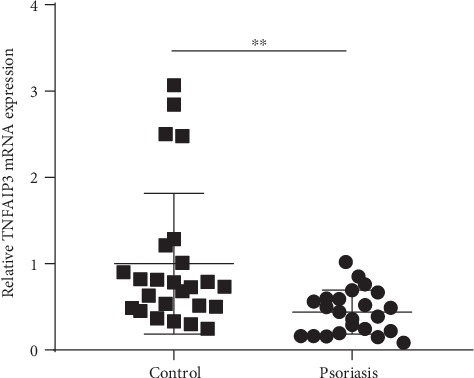
The relative *TNFAIP3* mRNA expression in patients with psoriasis and healthy controls. The relative *TNFAIP3* mRNA expression in PBMC of patients with psoriasis and healthy control was detected by RT-PCR. The *TNFAIP3* expression in psoriasis was much lower than that in healthy control. ^∗∗^*P* < 0.01.

To investigate the role of *TNFAIP3* in psoriasis, we collected PBMCs from patients with psoriasis and healthy controls. The mRNA expressions of *TNFAIP3* were measured by RT-PCR. The study included 23 moderate-to-severe psoriatic patients aged between 15 and 65 years (39.13 ± 14.56) and 25 healthy volunteers aged between 23 and 58 years (39.12 ± 10.03). The clinical characteristics of patients with psoriasis and healthy controls included in our study are summarized in Table 1. *TNFAIP3* mRNA expression in PBMCs was significantly lower in patients with psoriasis (mean ± SD = 0.44 ± 0.25) compared with normal subjects (mean ± SD = 1.00 ± 0.82; *P* < 0.01; Figure 1).

### 3.2. Establishment of an Imiquimod-Induced Psoriasis-Like Dermatitis Model in BALB/c Mice

**Figure 2:**
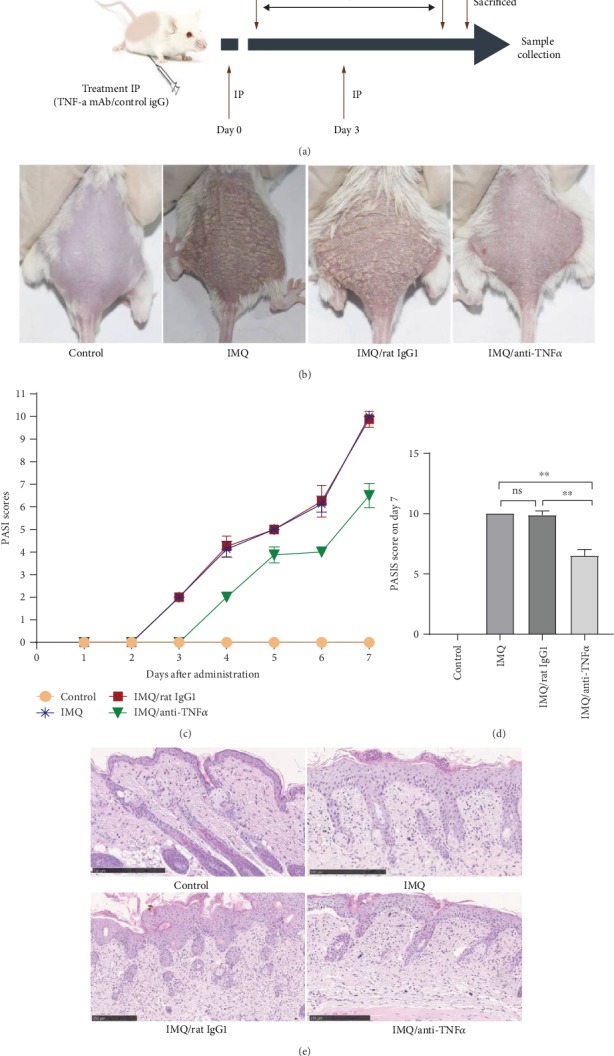
Establishment of an imiquimod-induced psoriasis-like dermatitis model in BALB/c mice and the effect of TNF-*α* antagonists: (a) schematic representation of the experimental plan of with TNF-*α* antagonists or control rat IgG1 on IMQ-treated mice; (b) representative pictures of IMQ-induced psoriasis-like cutaneous lesions from each group; (c) cumulative scores of the back skin were calculated from each group daily; (d) PASI score in day 7 after IMQ treatment. ^∗∗^*P* < 0.01; (e) representative hematoxylin and eosin staining of skin sections from each group on day 7 (bar = 250 mm).

To further explore the role of *TNFAIP3* in the pathogenesis of psoriasis, we established an IMQ-induced psoriasis-like dermatitis model, which is widely used as a rodent model of psoriasis. In accordance with previous studies, BALB/c mice were treated with 5% imiquimod cream for 6 days and examined daily using the modified PASI system. Anti-TNF-*α* antibody or control rat IgG1 was administered 1 day before (day −1) and 3 days after imiquimod treatment (day 3) as shown in Figure 2(a). Subsequently, erythema, scaling, and thickness of the dorsal skin were induced in the model group (Figure 2(b)), and these symptoms were attenuated in the IMQ/anti-TNF-*α* group. As shown in Figure 2(c), accumulative PASI scores were scored daily and the score at day 7 in the IMQ group was 10 ± 0 g compared with a score of 9.87 ± 0.35 g in the IMQ/rat IgG1 group (*P* > 0.05). The PASI score was decreased in the IMQ/anti-TNF-*α* group on day 7 after IMQ treatment (mean ± SD = 6.5 ± 0.53 g) (Figure 2(d)). Next, histological examination was performed to evaluate skin inflammation. The control group showed no obvious pathological changes in the skin. The IMQ group and IMQ/rat IgG1 groups revealed parakeratosis, Munro's microabscesses, extended acanthosis, and inflammatory cell infiltration, but anti-TNF-*α* antibody treatment reduced the thickness of the epidermis layer (Figure 2(e)). These data indicate that IMQ-treated skin lesions exhibited features typical of human psoriatic skin, but that anti-TNF-*α* antibody treatment suppressed IMQ-induced psoriasiform dermatitis.

### 3.3. *TNFAIP3* mRNA Expression Is Decreased in IMQ-Treated Mice

**Figure 3:**
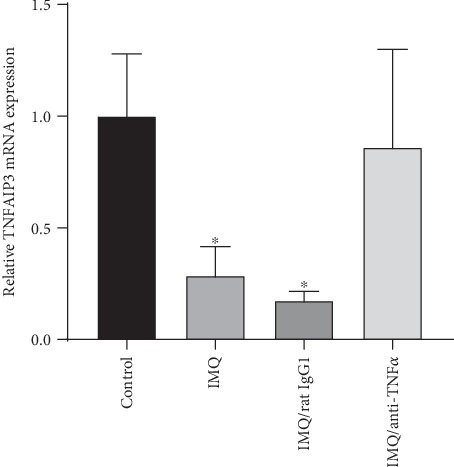
The relative *TNFAIP3* mRNA expression in PBMC from each group of IMQ-treated mice. *TNFAIP3* mRNA expression decreased in IMQ-treated mice as compared to the control group and increased by the treatment with TNF-*α* antagonists. ^∗^*P* < 0.05 compared to the control group.

To investigate the role of *TNFAIP3* in the IMQ-induced psoriasis-like dermatitis model, we collected PBMCs from mice and detected *TNFAIP3* mRNA expression by RT-PCR. The application of IMQ downregulated *TNFAIP3* expression (mean ± SD of the relative *TNFAIP3* mRNA expression: 1.00 ± 0.28 in the control group; 0.29 ± 0.13 in the IMQ group; 0.17 ± 0.04 in the IMQ/rat IgG1 group; and 0.86 ± 0.44 in the IMQ/anti-TNF-*α* group; *P* < 0.05) (Figure 3). Moreover, *TNFAIP3* expression in the IMQ/anti-TNF-*α* group was upregulated after treatment with TNF-*α* antagonists.

### 3.4. Th17- and Th1-Related Inflammatory Cytokines Are Increased in IMQ-Treated Mice

**Figure 4:**
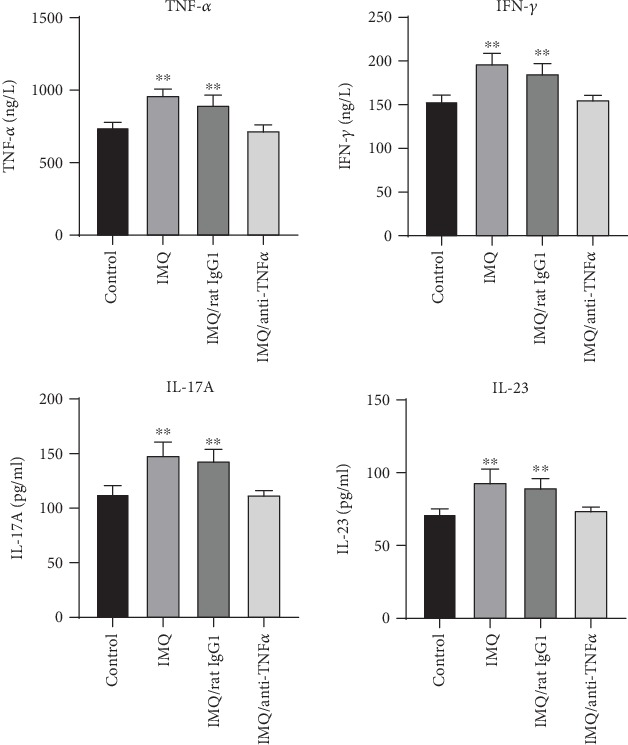
The serum levels of IFN- *γ*, IL-17A, TNF-*α*, and IL-23 were examined using ELISA. The data are presented as the mean ± SD. ^∗∗^*P* < 0.01 compared to the control group.

Serum levels of IFN-*γ*, IL-17A, IL-23, and TNF-*α* were measured by ELISA, and the results are summarized in Table 2. As shown in Figure 4, the levels of Th17 cytokines (IL-17A and IL-23) and Th1 cytokines (IFN-*γ* and TNF-*α*) in the serum were significantly higher in the IMQ group and IMQ/rat IgG1 group compared with the control group (*P* < 0.01). The application of TNF-*α* antagonists significantly decreased the levels of inflammatory cytokines in the IMQ-induced psoriasis-like dermatitis model.

### 3.5. p38 MAPK Is Activated in IMQ-Treated Mice

**Figure 5:**
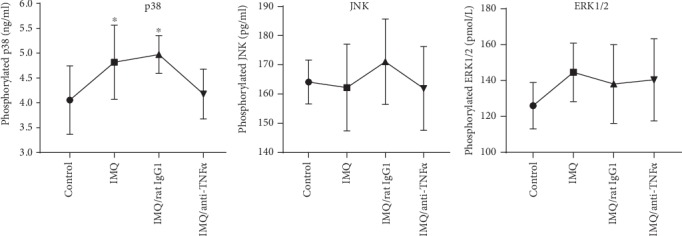
The phosphorylation levels of MAPK (p38, JNK, and ERK1/2) in serum were detected using ELISAs. The data are presented as the mean ± SD. ^∗^*P* < 0.05 compared to the control group.

Activation of the MAPK signaling pathway contributes to the regulation of the production of inflammatory mediators and Th17 cell differentiation. We examined the phosphorylation levels of p38, JNK, and ERK1/2 in mouse serum by ELISA. The phosphorylation of p38 was increased in the IMQ group and IMQ/rat IgG1 group compared with the control group but was downregulated after treatment with TNF-*α* antagonists (mean ± SD of p38 phosphorylation: 4.05 ± 0.69 ng/ml in the control group; 5.03 ± 0.60 ng/ml in the IMQ group; 4.97 ± 0.38 ng/ml in the IMQ/rat IgG1 group; and 4.17 ± 0.50 ng/ml in the IMQ/anti-TNF-*α*; *P* < 0.05) (Figure 5). However, the phosphorylation levels of ERK1/2 and JNK among the four groups were similar.

### 3.6. Th1 and Th17 Cells Are Increased in IMQ-Treated Mice

**Figure 6:**
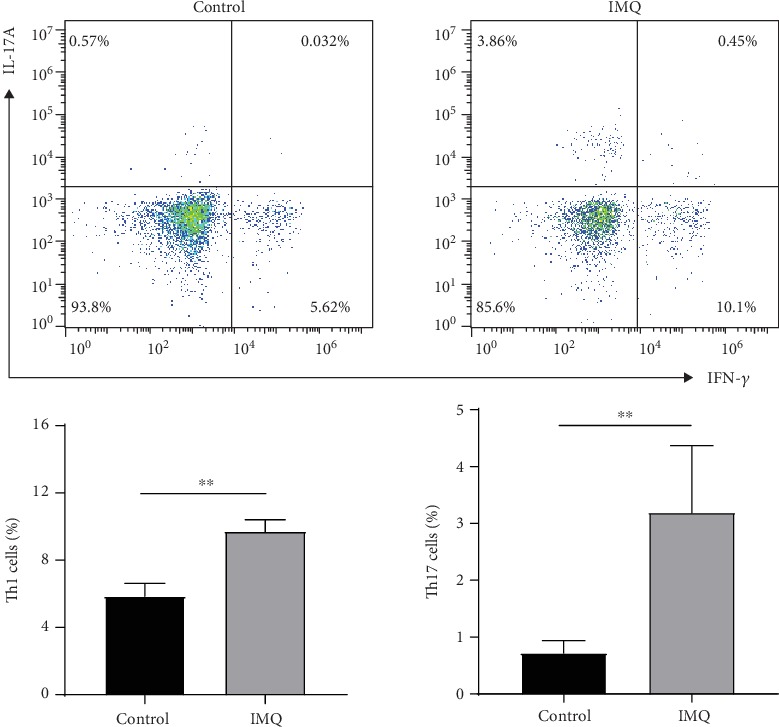
The proportion of Th1 and Th17 cells in the spleen from the IMQ group and control group. Th1 cells and Th17 cells significantly increased in the IMQ group compared with the control group (*P* < 0.01). ^∗∗^*P* < 0.01 compared to the control group.

To confirm Th1 and Th17 cell differentiation in IMQ treated mice, the proportions of Th1 and Th17 cells in spleens were analyzed by flow cytometry. The proportion of Th1 and Th17 cells was significantly increased in the IMQ group compared with the control group (*P* < 0.01) (Figure 6).

## 4. Discussion

In this study, we found that *TNFAIP3* expression was decreased in the PBMCs of psoriatic patients and confirmed its role in association with Th1 and Th17 cell differentiation and p38 activation in psoriasis using the IMQ-induced psoriasis-like dermatitis model. Furthermore, TNF-*α* antagonist treatment alleviated skin inflammation caused by imiquimod application.

Psoriasis is a chronic T cell-mediated inflammatory skin disease. Previous studies reported that Th1 and Th17 cells have critical roles in the pathogenesis of psoriasis [16]. Inflammatory cytokines, including IL-17, IFN-*γ*, and TNF-*α*, are also involved in the inflammatory process of psoriasis [17]. Taken together, the pathogenesis of psoriasis involves complicated immune regulation with Th1 and Th17 dysregulation. Studies of immune regulation in psoriasis provide new insights regarding psoriasis. *TNFAIP3* had an important effect in several inflammatory diseases, such as systemic lupus erythematosus, inflammatory bowel disease, coeliac disease, and psoriasis [18]. Previously, it was shown that *TNFAIP3* polymorphisms were associated with susceptibility to psoriasis [19]. Here, we demonstrated that *TNFAIP3* mRNA was expressed at low levels in psoriatic patients and the IMQ-induced psoriasis-like dermatitis model and that TNF-*α* antagonists increased *TNFAIP3* expression and attenuated the severity of psoriasis in the mouse model. This finding was in accordance with the findings of Sahlol et al., who reported reduced *TNFAIP3* expression in psoriatic skin and blood of patients with psoriasis [20]. However, in contrast to our results, Liu et al. showed that TNFAIP3 protein expression in lesional skin was increased under psoriatic inflammation. The authors explained that increased TNFAIP3 protein expression might be induced by high levels of TNF-*α* in psoriatic skin and a feedback mechanism involving the NF-*κ*B pathway [21]. Furthermore, our data support the idea that the antipsoriatic effect of TNF-*α* antagonists might be mediated by *TNFAIP3* regulation.

Our results showed that numbers of Th1 and Th17 cells and their related inflammatory cytokines were increased under psoriatic inflammation, which were associated with the decreased *TNFAIP3* mRNA expression. Of note, cytokine expression decreased after treatment with TNF-*α* antagonists. Similarly, other studies reported that the severity of skin inflammation in epidermis-specific *TNFAIP3*-knockout mice was exacerbated and associated with the upregulation of proinflammatory cytokines and chemokines [7]. Another study showed that *TNFAIP3* deficiency by siRNA silencing or in knockout cells led to enhanced IL-17-dependent inflammatory gene expression, which indicates that *TNFAIP3* might be an inhibitor of IL-17 signaling [9]. Therefore, the decreased *TNFAIP3* expression in psoriasis might be associated with the increase in Th1 and Th17 cell numbers and their related cytokines. These results suggest a new mechanism involved in immune dysfunction in psoriasis. However, in addition to Th1 and Th17 cells, many other immune cells, such as dendritic cells and macrophages, participate in psoriasis development. Therefore, whether *TNFAIP3* regulates other immune cells and cytokines requires further research.

In addition to the function of *TNFAIP3* as a regulator of the NF-*κ*B pathway, there is evidence that *TNFAIP3* is also an important regulator of other immune pathways including MAPK signaling [22]. Garg et al. showed that TNFAIP3 suppressed activation of the MAPK pathway [23]. Additionally, p38 MAPK activation was essential for IL-17 production by CD4^+^ T cells in an allergic encephalomyelitis model [5]. Furthermore, the silencing of TNFAIP3 increased JNK and p38 activation but did not affect ERK1/2 in Behcet's disease [24]. To further clarify the underlying mechanisms involved in MAPK signaling, we assessed MAPK activation in psoriasis and its relationship with the effects of anti-TNF-*α* therapy. Our data suggest that p38, but not ERK1/2 and JNK, was phosphorylated after mice were treated with IMQ. Furthermore, pretreatment with anti-TNF-*α* therapy significantly inhibited the activation of p38. Therefore, it is not surprising that TNFAIP3 downregulation is accompanied by p38 MAPK activation under psoriatic inflammation.

It was previously demonstrated that TNF-*α* antagonists have high efficacy for the treatment of psoriasis by inhibiting the inflammation cascade triggered by TNF-*α* [25]. Studies also reported that anti-TNF therapy interfered with immune responses to modulate the productions of IFN-*γ*, TNF-*α*, and IL-17A [17]. However, the exact mechanisms involved in the effects of pathogenic cytokines in psoriasis and antipsoriatic treatment with TNF-*α* antagonists remain unclear. Our data support the idea that TNF-*α* antagonists attenuate skin inflammation and increase *TNFAIP3* expression accompanied by the downregulation of Th1- and Th17-related cytokines and phosphorylated p38 levels. Therefore, TNF-*α* antagonists might exert effects on psoriasis via this pathway.

## 5. Conclusions

In summary, *TNFAIP3* expression was downregulated in psoriasis, which might be associated with Th1 and Th17 cell differentiation and p38 activation. Furthermore, the anti-inflammatory effect of anti-TNF-*α* therapy involved this pathway. These results demonstrate that *TNFAIP3* might be a potential new target in the research of immune dysfunction in psoriasis. Future studies should focus on the precise mechanism by which *TNFAIP3* regulates the interaction between inflammatory cytokines and other immune cells.

## Figures and Tables

**Table 1:** Baseline demographics of patients with psoriasis and healthy controls.

Characteristics	Psoriasis (*n* = 23)	Health controls (*n* = 25)
Age, mean ± SD (range) (years)	39.13 ± 14.56 (15-65)	39.12 ± 10.03 (23-58)
Male/female	12/11	10/15
Family history of psoriasis, *n* (%)	4 (17.39%)	0
Duration (years)	14.07	0

**Table 2:** Comparison of cytokine levels among the four groups (mean ± SD).

	Control group (*n* = 8)	IMQ group (*n* = 7)	IMQ/rat IgG1 group (*n* = 7)	IMQ/anti-TNF-*α* group (*n* = 8)	*P* value
TNF-*α* (ng/l)	744.00 ± 34.55	962.71 ± 44.46	895.12 ± 70.37	719.97 ± 40.96	*P* < 0.01
IFN-*γ* (ng/l)	154.12 ± 6.94	196.81 ± 12.14	185.45 ± 11.51	155.74 ± 4.98	*P* < 0.01
IL-17A (pg/ml)	114.07 ± 7.78	151.99 ± 6.80	143.09 ± 10.90	112.12 ± 3.97	*P* < 0.01
IL-23 (pg/ml)	72.00 ± 3.48	96.05 ± 5.28	89.70 ± 6.31	74.05 ± 2.39	*P* < 0.01

## Data Availability

The statistically analyzed data used to support the findings of this study are included within the article. The raw data used to support the findings of this study are available from Hongzhong Jin, the corresponding author, upon request.
